# Enzalutamide therapy for advanced prostate cancer: efficacy, resistance and beyond

**DOI:** 10.1530/ERC-18-0289

**Published:** 2018-09-14

**Authors:** Simon Linder, Henk G van der Poel, Andries M Bergman, Wilbert Zwart, Stefan Prekovic

**Affiliations:** 1 Division of OncogenomicsOncode Institute, The Netherlands Cancer Institute, Amsterdam, The Netherlands; 2 Division of UrologyThe Netherlands Cancer Institute, Amsterdam, The Netherlands; 3 Division of Medical OncologyThe Netherlands Cancer Institute, Amsterdam, The Netherlands; 4 Division of OncogenomicsThe Netherlands Cancer Institute, Amsterdam, The Netherlands; 5 Laboratory of Chemical Biology and Institute for Complex Molecular SystemsDepartment of Biomedical Engineering, Eindhoven University of Technology, Eindhoven, The Netherlands

**Keywords:** enzalutamide, treatment resistance, biomarkers, androgen receptor, mutations, prostate cancer, mCRPC, androgen deprivation therapy, docetaxel

## Abstract

The androgen receptor drives the growth of metastatic castration-resistant prostate cancer. This has led to the development of multiple novel drugs targeting this hormone-regulated transcription factor, such as enzalutamide – a potent androgen receptor antagonist. Despite the plethora of possible treatment options, the absolute survival benefit of each treatment separately is limited to a few months. Therefore, current research efforts are directed to determine the optimal sequence of therapies, discover novel drugs effective in metastatic castration-resistant prostate cancer and define patient subpopulations that ultimately benefit from these treatments. Molecular studies provide evidence on which pathways mediate treatment resistance and may lead to improved treatment for metastatic castration-resistant prostate cancer. This review provides, firstly a concise overview of the clinical development, use and effectiveness of enzalutamide in the treatment of advanced prostate cancer, secondly it describes translational research addressing enzalutamide response vs resistance and lastly highlights novel potential treatment strategies in the enzalutamide-resistant setting.

## Introduction

**Figure 1:**
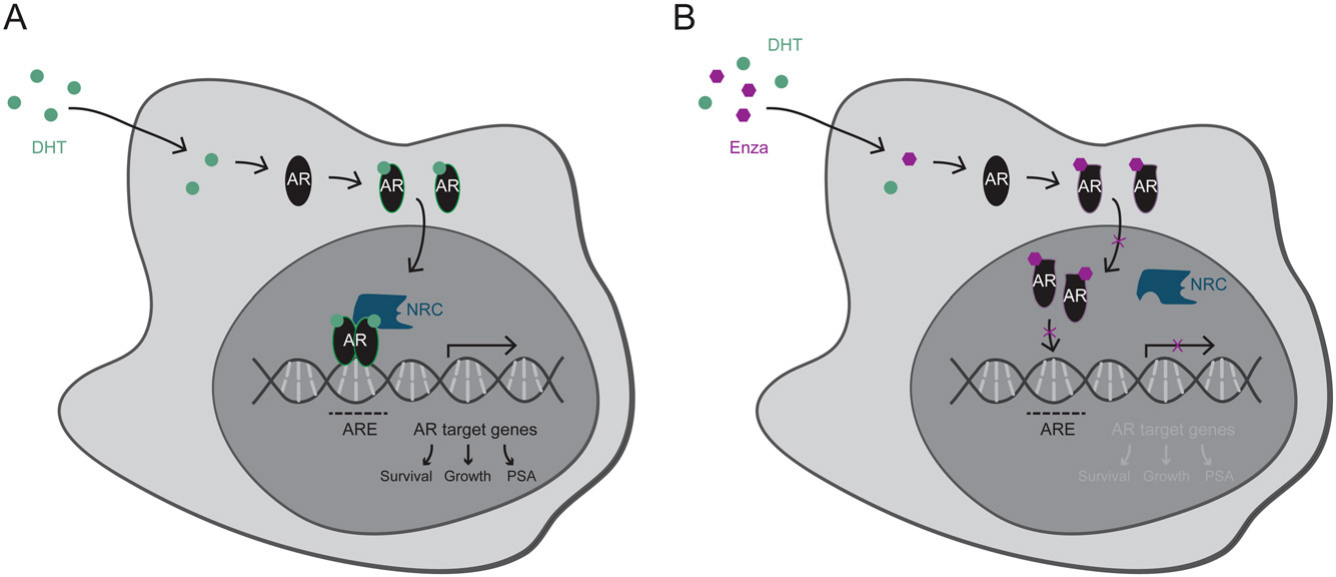
AR signaling axis and mechanism of action of enzalutamide. (A) Upon dihydrotestosterone (DHT) binding, the AR dimerizes and translocates to the nucleus, where it binds to AR-response elements (ARE) and recruits nuclear receptor coregulators (NRC), so-called coactivators or corepressors, to regulate transcription of directly responsive genes involved in cell proliferation and survival. (B) Enzalutamide (Enza) binding to the ligand-binding pocket of the AR results in a conformational change, rendering the receptor incapable of forming an active transcriptional complex. Further, enzalutamide blocks AR nuclear translocation and the enzalutamide-bound AR is impaired in its DNA-binding ability, ultimately preventing AR-dependent gene expression.

Ever since the discovery that prostate cancer (PCa) growth after androgen deprivation therapy (ADT) remains dependent on androgen receptor (AR) signaling, researchers have been looking for new effective ways to block the action of this hormone-dependent transcription factor (Attard *et al.* 2009, Tran *et al.* 2009, Scher *et al.* 2012). Upon stimulation with androgens, the AR dissociates from its molecular chaperones and translocates to the nucleus, where it binds to thousands of sites throughout the human genome to regulate transcription of directly responsive genes, including pro-mitotic genes involved in tumor cell proliferation (Fig. 1A) (Brinkmann *et al.* 1999, Itkonen & Mills 2012, Mills 2014).

Inhibiting androgen signaling through ADT initially results in tumor regression in the vast majority of cases, but inevitably the tumor cells adapt to low androgen levels, leading to disease progression, which is known as castration resistance (Harris *et al.* 2009, Massard & Fizazi 2011, Karantanos *et al.* 2013).

Potent antiandrogens, that either target the AR directly through physical competition with the receptor’s natural ligand dihydrotestosterone (DHT) or indirectly via inhibition of androgen biosynthesis, are among the treatment options for metastatic castration-resistant prostate cancer (mCRPC) (Helsen *et al.* 2014).

At the moment, enzalutamide (MDV-3100) is the most frequently prescribed compound for treatment of mCRPC (Sanford 2013). This drug belongs to the class of direct androgen receptor inhibitors and tackles the AR pathway at multiple nodes: by preventing ligand binding, by blocking AR nuclear translocation and by inhibiting DNA transactivation, ultimately abrogating the expression of androgen-responsive genes (Fig. 1B) (Tran *et al.* 2009, van Soest *et al.* 2013). The multiple stage actions of enzalutamide on AR signaling are considered the main reason for its superior clinical activity over other direct AR inhibitors, such as flutamide, bicalutamide and nilutamide (Antonarakis 2013).

However, due to inter-patient heterogeneity of PCa, which is widely recognized as a major drawback for therapy efficacy, treatment responses to enzalutamide vary between patients (Boyd *et al.* 2012). Whereas some patients do not have a substantial clinical benefit from enzalutamide therapy, others who do benefit, start progressing after a certain period of time, which is also dependent on therapy sequencing (Scher *et al.* 2012, Beer *et al.* 2014, Merseburger *et al.* 2015).

**Figure 2:**
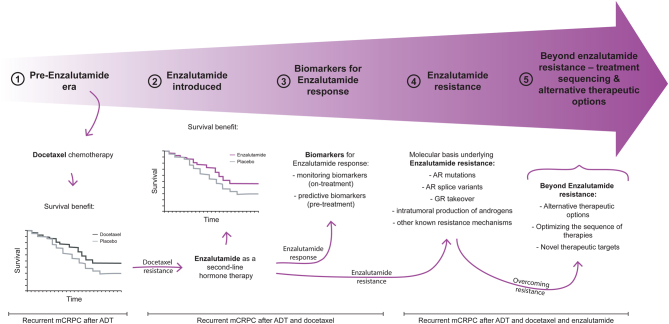
Graphical summary capturing the topics discussed in this review. Docetaxel has been the first agent showing a survival benefit in mCRPC patients (1). Despite initial responses upon docetaxel chemotherapy, patients eventually progress, whereby enzalutamide has been shown to be effective in such a docetaxel-resistant mCRPC setting (2). Current translational research efforts are aimed at developing biomarkers for enzalutamide response (3), understanding molecular underpinnings of enzalutamide-resistant mCRPC (4) and optimizing treatment strategies to overcome enzalutamide resistance (5).

This review, of which the content is illustrated in Fig. 2 (1–5), will firstly provide a comprehensive insight into the use of enzalutamide in the treatment of advanced PCa – spanning from treatment options in the pre-enzalutamide era (1) to its preclinical development and the landmark studies that led to its FDA approval for mCRPC (2). Thereupon, we discuss translational research directed at tackling unmet clinical needs in the treatment of advanced PCa using enzalutamide. This includes having on-treatment and predictive biomarkers for treatment response (3); a better understanding of molecular mechanisms underlying enzalutamide resistance (4); and lastly, the development of novel therapeutic approaches aimed to overcome therapy resistance (5).

## The pre-enzalutamide era

### Androgen deprivation therapy

ADT has been the standard of care for patients with symptomatic metastatic PCa since the forties of the last century (Merseburger *et al.* 2016). However, despite initial response to ADT, eventually resistance emerges in practically every patient, which is mediated by AR-dependent or -independent pathways (Scher & Sawyers 2005). Initially, two retrospective studies have shown a limited survival benefit of continued androgen suppression with luteinizing hormone-releasing hormone (LHRH) analogs in the mCRPC setting (Taylor *et al.* 1993, Hussain *et al.* 1994). Based on these findings, all mCRPC patients enrolled in the trials discussed further below continue androgen suppression therapy. Although data are limited, the benefits of continuing androgen deprivation outweighed the potential risks of discontinuing the therapy.

### Chemotherapy

In 2004, the TAX-327 trial initiated a transition in systemic mCRPC treatment (Tannock *et al.* 2004). In this phase III study, 1006 patients with mCRPC were randomized to receive prednisone either in combination with mitoxantrone (a chemotherapy that provides palliation, but does not lead to an improvement in survival for patients with castration-refractory PCa (Tannock *et al.* 1996) or with docetaxel (a chemotherapy that has been reported in phase II studies to successfully reduce serum prostate-specific antigen (PSA) levels Beer *et al.* 2001, Berry *et al.* 2001).

Whereas mitoxantrone, as a type II topoisomerase inhibitor that intercalates between DNA bases and thereby disrupts DNA synthesis and repair (Nitiss 2009, Pommier *et al.* 2010), is not directly linked to AR biology, docetaxel is. It belongs to the taxane class of chemotherapeutic agents that bind to tubulin and hyperstabilize microtubules, which ultimately leads to impairments of the mitotic cell cycle and AR signaling by preventing its nuclear translocation (Kuroda *et al.* 2009, Zhu *et al.* 2010, Darshan *et al.* 2011, Fitzpatrick & de Wit 2014).

The TAX-327 study identified docetaxel as the first chemotherapeutic drug that showed a modest overall survival (OS) benefit compared to mitoxantrone (Table 1) (Berthold *et al.* 2008). Based on these results, docetaxel was established as a first-line therapy option for both, symptomatic as well as asymptomatic mCRPC.

**Table 1:** Clinical trials of systemic treatments for mCRPC that improve overall survival.

	Trial (registration number)	Study intervention		Median overall survival (95% CI)		Hazard ratio (95% CI; *P*-value)	References	Sequence
		Treatment	Control	Treatment	Control			
Chemotherapy	TAX-327^a^	Docetaxel + Prednisone	Mitoxantrone + Prednisone	19.2 months (17.5–21.3)	16.3 months (14.3–17.9)	0.76 (0.62–0.94; *P* = 0.009)	Berthold *et al*. (2008), Tannock *et al*. (2004)	Progression after ADT without chemotherapy
Hormonal therapy	PREVAIL (Nbib1212991)	Enzalutamide	Placebo	35.3 months (32.2–not yet reached)	31.3 months (28.8–34.2)	0.77 (0.67–0.88; *P* = 0.0002)	Beer *et al*. (2017, 2014)	Progression after ADT without chemotherapy
	AFFIRM (Nbib974311)	Enzalutamide	Placebo	18.4 months (17.3–not yet reached)	13.6 months (11.3–15.8)	0.63 (0.53–0.75; *P* < 0.001)	Scher *et al*. (2012)	Progression after ADT and docetaxel

^a^No trial registration number available for TAX-327.

ADT, androgen-deprivation therapy; CI, confidence interval.

### Docetaxel resistance

As described earlier, mCRPC patients treated with docetaxel-based chemotherapy have a modest OS benefit implying most patients will progress rather rapidly. In patients with a good initial response to docetaxel therapy, re-challenging with the same chemotherapeutic agent results in a PSA response in up to 60% of patients with a median time to progression of 6 months (Beer *et al.* 2004). As this response is less profound as compared to the therapeutic effect in the first round, it could also be hypothesized that the efficacy of docetaxel re-challenge will keep decreasing until its effect becomes negligible. Mechanisms underlying this docetaxel resistance in the mCRPC setting can be diverse (Seruga *et al.* 2011). On the one hand, those include rather general mechanisms associated with resistance to taxanes, including an altered microtubule composition affecting docetaxel binding (such as upregulation of certain isotypes Ranganathan *et al.* 1998 or mutations Yin *et al.* 2010), a reduced intracellular drug accumulation due to overexpression of drug efflux pumps (such as P-glycoprotein Zhu *et al.* 2013) or an impaired drug distribution due to aberrant angiogenesis (Marignol *et al.* 2008). On the other hand, resistance can also develop due to mechanisms intrinsic to the biology of mCRPC like continued AR signaling which stimulates PCa growth and inhibits apoptosis (Seruga *et al.* 2011) or due to the activation of compensatory oncogenic pathways (such as PI3K/AKT or MAPK/ERK Zhu & Kyprianou 2008) which are themselves associated with proliferation and survival. As a result of taxane resistance, new therapeutic approaches tackling docetaxel-resistant mCRPC were needed and much sought-after.

## Enzalutamide as a second-line hormone therapy

### Preclinical development

Ever since molecular profiling studies have revealed that many CRPC tumors remain AR driven, there has been great interest in identifying novel and potent strategies to better block the AR signaling axis (Chen *et al.* 2004). Such next-generation antiandrogens should – unlike their first-generation counterparts (e.g. bicalutamide and flutamide) – preferably possess greater AR-binding affinities without any agonistic effects (Chen *et al.* 2004, Bambury & Scher 2015). In their search for such improved antiandrogens, Tran *et al.* (2009) screened nearly 200 thiohydantoin derivatives of RU59063 – a non-steroidal AR agonist with a relatively high affinity and selectivity over other nuclear hormone receptors – for retained activity in human PCa cells that overexpressed the AR protein, which is also clinically observed in the castration-resistant disease setting. RD162 and MDV3100 (now enzalutamide) were selected as the lead compounds for additional biological validation, and importantly, both antiandrogens led to tumor regression in xenograft models (Tran *et al.* 2009). Due to its favorable drug-like properties, such as oral bioavailability and longer serum half-life, enzalutamide was selected for further clinical development (Bambury & Scher 2015).

### Clinical testing

The preclinically demonstrated antitumor activity of enzalutamide was subsequently validated in a phase I/II trial, in which patients with progressive mCRPC were enrolled in dose-escalation cohorts, ultimately demonstrating its safety and tolerability, along with antitumor effect at all tested doses (Scher *et al.* 2010).

In 2012, the preliminary analysis of the AFFIRM trial was published, being the first phase III study on enzalutamide in the mCRPC setting (Scher *et al.* 2012). In this trial, 1199 mCRPC patients who progressed on docetaxel therapy were randomized to receive either enzalutamide or placebo. Enzalutamide treatment significantly improved patient outcome after docetaxel therapy compared to the placebo control group (Table 1).

The efficacy of enzalutamide and its limited toxicity as compared to chemotherapy could not only be achieved in mCRPC patients who were previously treated with docetaxel, but also in the chemotherapy-naïve setting, as addressed by the PREVAIL study (Beer *et al.* 2014). This was a randomized phase III trial including 1717 chemo-naïve mCRPC men comparing enzalutamide therapy to a placebo. Again, enzalutamide therapy resulted in a significant improvement in OS and radiographic progression-free survival (rPFS) (Table 1) (Beer *et al.* 2017).

Moreover, the results of the randomized phase III PROSPER trial were recently published. Therein, the addition of enzalutamide or placebo to continued ADT was tested with regards to its potential to delay metastasis formation in men with non-metastasized CRPC who are at high risk for developing distant lesions. In this setting, enzalutamide therapy led to a 71% lower risk of metastasis or death compared to placebo (Hussain *et al.* 2018).

Based on these results, enzalutamide is now a primary treatment option for metastasis-free CRPC and asymptomatic mCRPC, whereas docetaxel is mainly used in men with symptomatic metastasized disease and acquired resistance to first-line therapeutics (Ryan *et al.* 2013, Beer *et al.* 2014, Hussain *et al.* 2018).

## Biomarkers for enzalutamide response

The readout of PSA levels as a diagnostic biomarker was already introduced in the 1980s, but has also been questioned since then, mainly due to its non-specificity as a marker for cancerous lesions (Oesterling 1991, Salman *et al.* 2015). However, PSA measurements as a monitoring biomarker for either treatment response or resistance following PCa diagnosis and corresponding interventions, are routinely used in the clinic. PSA declines of at least 30% after 4 weeks and >30% or >50% after 12 weeks of treatment have been shown to correlate with a survival advantage especially in patients treated with AR-targeting compounds, whereas stable or increased PSA levels correlated with poorer outcome (Scher *et al.* 2008, Brasso *et al.* 2015, Fuerea *et al.* 2016, Rescigno *et al.* 2016). Moreover, circulating tumor cells (CTCs) seem to be a promising tool to predict a treatment-induced survival benefit. It has been observed that patients with a decline in number of CTCs (>30%) after 4 weeks of therapy have a better prognosis (Scher *et al.* 2015, Lorente *et al.* 2016, Heller *et al.* 2017, Prekovic *et al.* 2018*a*). Consequently, CTCs could be a better marker for treatment resistance in tumors progressing without an obvious PSA rise, taking into account that further validation is warranted before it can be recommended in daily clinical practice. These on-treatment readouts, however, solely allow monitoring of a patient’s response to for example, enzalutamide therapy. Whereas some men do respond exceptionally well and continue treatment for several years, others progress within months or even do not show any response at all (Attard & Antonarakis 2016). Thus, biomarkers that enable the identification of patient subpopulations that benefit from enzalutamide treatment are urgently needed to improve the management of PCa patients.

Especially in the primary disease setting, tissue biopsies have proved to be highly informative. Besides classification systems based on clinical parameters (such as Gleason score, PSA and clinical staging) (D’Amico *et al.* 1998), genomic analyses may provide risk-assessment biomarkers that stratify patients with PCa on outcome (Irshad *et al.* 2013, Knezevic *et al.* 2013, Stelloo *et al.* 2015). However, the bone-predominant metastatic landscape of CRPC renders them rather impractical in routine clinical practice and current approaches almost exclusively focus on minimally invasive biomarkers from blood (Wyatt *et al.* 2016). Until now, several studies have shown that the profiling of CTCs or cell-free tumor DNA (cfDNA) in liquid biopsies enables the detection of AR splice variants, *AR* copy number gains and *AR* mutations, all of whom are at least associated with enzalutamide resistance and poorer prognosis (Schwarzenbach *et al.* 2009, Antonarakis *et al.* 2014, Diaz & Bardelli 2014, Salvi *et al.* 2016, Wyatt *et al.* 2016, Conteduca *et al.* 2017). Nonetheless, no such biomarker is implemented and routinely used in the clinic thus far, and further studies that robustly validate each biomarker in a prospective fashion are required for a potential practice change (Attard & Antonarakis 2016).

## Molecular basis underlying enzalutamide resistance

The AFFIRM and PREVAIL trials clearly demonstrated the advantages of enzalutamide treatment. However, 46 (AFFIRM) and 22% (PREVAIL) of patients with mCRPC did not respond to second- or first-line treatment with enzalutamide, meaning that their PSA levels did not decline ≥50% from baseline. The remaining 54 and 78% of enzalutamide-treated patients responded initially, but PSA progression could be observed after a median time of 8.3 months (AFFIRM) and 11.2 months (PREVAIL) (Scher *et al.* 2012, Beer *et al.* 2014).

The mechanisms underlying this pre-existent or acquired resistance to enzalutamide are still not fully elucidated, but several possible mechanisms have been proposed (Claessens *et al.* 2014). In the next section, we will briefly discuss such potential mechanisms, which are elaborately discussed in Prekovic *et al.* (2018*b*).

### AR mutations

Gain-of-function mutations in the *AR* gene, especially within the exon 7 (encoding for the ligand-binding domain), have been found in 5–30% of CRPC patients (Taplin *et al.* 1999, Coutinho *et al.* 2016, Kumar *et al.* 2016, Rathkopf *et al.* 2017, Pal *et al.* 2018). These genomic alterations do not only permit receptor activation by various circulating steroids next to testosterone (such as H875Y or T878A), but may also alter the responsiveness of the AR to antiandrogens, resulting in antagonist-to-agonist switching (Grossmann *et al.* 2001, Nadal *et al.* 2017, Prekovic *et al.* 2018*a*). This is exemplified by the F877L/T878A and M896V/S889G double mutants, which were associated with resistance (Lallous *et al.* 2016, Prekovic *et al.* 2016) and have recently been found in cfDNA extracted from plasma of mCRPC patients progressing on enzalutamide therapy (Azad *et al.* 2015, Wyatt *et al.* 2016).

### AR splice variants

Alternatively spliced AR variants, especially AR-V7, have been reported to be implicated in resistance to AR-targeting drugs, including enzalutamide. AR-V7 is an AR isoform that lacks the ligand-binding domain (LBD), causing the variant to be constitutively active and resistant to LBD-targeting inhibitors (Guo *et al.* 2009, Hu *et al.* 2009, Antonarakis *et al.* 2016). Multiple studies have demonstrated that AR-V7 expression is a biomarker for resistance to AR-targeting drugs in CRPC (Li *et al.* 2013, Scher *et al.* 2016, Antonarakis *et al.* 2017, Del Re *et al.* 2017, Qu *et al.* 2017, Todenhofer *et al.* 2017), but it remains to date unclear whether AR-V7 is driving the resistance or whether it merely is a manifestation of treatment-induced selective pressure without being the key-driver to therapy failure.

### Glucocorticoid receptor takeover

The glucocorticoid receptor (GR) has been reported to be upregulated or re-expressed after AR blockade, indicating a complex cross-talk between AR and GR biology. Due to great similarities in the mechanism of action between nuclear receptors, GR is suggested to take over the role of AR by driving the expression of a subset of androgen-responsive genes, thus enabling the tumor to progress even in presence of the AR-selective antagonist enzalutamide (Arora *et al.* 2013, Kach *et al.* 2015, Li *et al.* 2017, Shah *et al.* 2017, Puhr *et al.* 2018).

### Intratumoral production of androgens

In addition, reactivation of the AR can occur via intratumoral production of androgens, enabling the prostate cancers to progress despite ongoing androgen deprivation (Locke *et al.* 2008). The expression of one of the essential enzymes in androgen biosynthesis, AKR1C3, was significantly increased in enzalutamide-resistant cells and xenograft tumors as well as in clinical specimens of advanced PCa, making it an attractive therapeutic target (Wako *et al.* 2008, Pfeiffer *et al.* 2011, Hamid *et al.* 2013, Liu *et al.* 2015, 2017). Inhibition of AKR1C3 as a novel therapeutic strategy is currently under investigation in a clinical trial (Nbib2935205) studying its potential benefit in combination with enzalutamide therapy in mCRPC (Pan *et al.* 2018).

### Other known resistance mechanisms

Next to the aforementioned AR-related underpinnings of enzalutamide resistance, several additional mechanisms have been described to give rise to therapy resistance, but are not within the scope of this review. Among those are very diverse adaptations, such as metabolic changes (e.g. shifting to aerobic glycolysis Cui *et al.* 2014 or alterations in the hexosamine biosynthetic pathway Itkonen *et al.* 2016), but also autophagy (Nguyen *et al.* 2014) or activation of certain signaling pathways (such as Wnt Lee *et al.* 2015 or interleukin 6 Liu *et al.* 2014*b*) – all of whom are addressed in depth in Prekovic *et al.* (2018*b*).

## Beyond enzalutamide resistance – therapy sequencing and alternative therapeutic options

**Figure 3:**
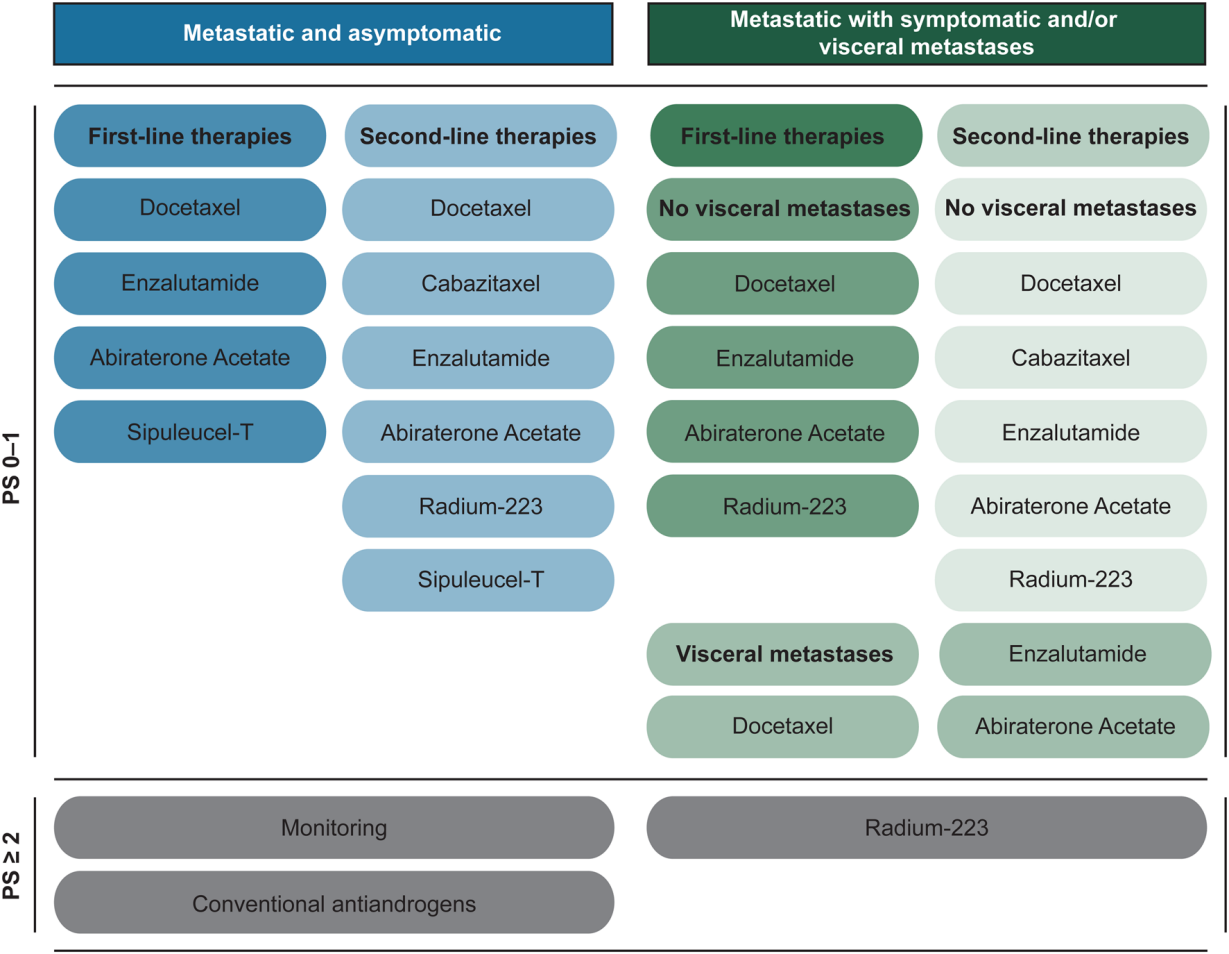
Schematic representation of treatment options in the mCRPC setting according to current standards of care. Therapeutic options are subdivided in first- and second-line therapies based on the clinical setting of the disease (asymptomatic vs symptomatic or visceral metastases). Treatment options are also determined by the overall performance status (PS) of the patient (PS 0–1: normal activity or some symptoms, but almost entirely ambulatory; PS ≥2: symptomatic patients <50% of daytime in bed up until completely bedridden).

Scheduling of enzalutamide treatment in mCRPC patients can differ greatly; depending on a patient’s PCa stage, overall health status, treatment history and personal preference (Fig. 3). The mechanisms behind enzalutamide resistance (or other AR antagonists) may therefore also differ as these may depend on the settings in which the drug was administered. Over the last decade, several treatments have been developed, even though the optimal sequence of therapies still remains to be determined. This is especially the case, since none of the available therapeutic options described in Table 2 have yet been compared head-to-head in clinical trials (Sartor & Gillessen 2014).

**Table 2:** Multiple large clinical trials of alternative therapies that improve survival of patients with mCRPC.

	Trial (registration number)	Study intervention		Median overall survival (95% CI)		Hazard ratio (95% CI; *P*-value)	References	Sequence
		Treatment	Control	Treatment	Control			
Hormonal therapy	COU-AA-302 (Nbib887198)	Abiraterone + Prednisone	Placebo + Prednisone	34.7 months (32.7–36.8)	30.3 months (28.7–33.3)	0.81 (0.70–0.93; *P* = 0.0033)	Ryan *et al*. (2013, 2015), Rathkopf *et al*. (2014)	Progression after ADT without chemotherapy
	COU-AA-301 (Nbib638690)	Abiraterone + Prednisone	Placebo + Prednisone	15.8 months (14.8–17.0)	11.2 months (10.4–13.1)	0.74 (0.64–0.86; *P* < 0.0001)	De Bono *et al*. (2011), Fizazi *et al*. (2012)	Progression after ADT and docetaxel
Chemotherapy	TROPIC (Nbib417079)	Cabazitaxel + Prednisone	Mitoxantrone + Prednisone	15.1 months (14.1–16.3)	12.7 months (11.6–13.7)	0.70 (0.59–0.83; *P* < 0.0001)	de Bono *et al*. (2010), Bahl *et al*. (2013)	Progression after ADT and docetaxel
Immunotherapy	IMPACT (Nbib65442)	Sipuleucel-T	Placebo	25.8 months (22.8–27.7)	21.7 months (17.7–23.8)	0.78 (0.61–0.98; *P* = 0.03)	Kantoff *et al*. (2010)	Progression after ADT, unspecified docetaxel status
Alpha-particle Therapy	ALSYMPCA (Nbib699751)	Radium-223	Placebo	14.9 months (13.9–16.1)	11.3 months (10.1–12.8)	0.70 (0.58–0.83; *P* < 0.001)	Parker *et al*. (2013)	Progression after ADT, unspecified docetaxel status

ADT, androgen-deprivation therapy; CI, confidence interval.

### Available therapeutic options in clinical practice

For mCRPC patients responding to enzalutamide, there is no doubt that outcomes have improved significantly. Nevertheless, despite the survival benefits, patients are still progressing and improvements in absolute survival rates are rather disappointing. Besides enzalutamide, several other therapeutic options with proven benefit for mCRPC patients have been developed in the past 10 years, which are summarized in Table 2 and will be briefly discussed hereafter.

#### Cabazitaxel

**Figure 4:**
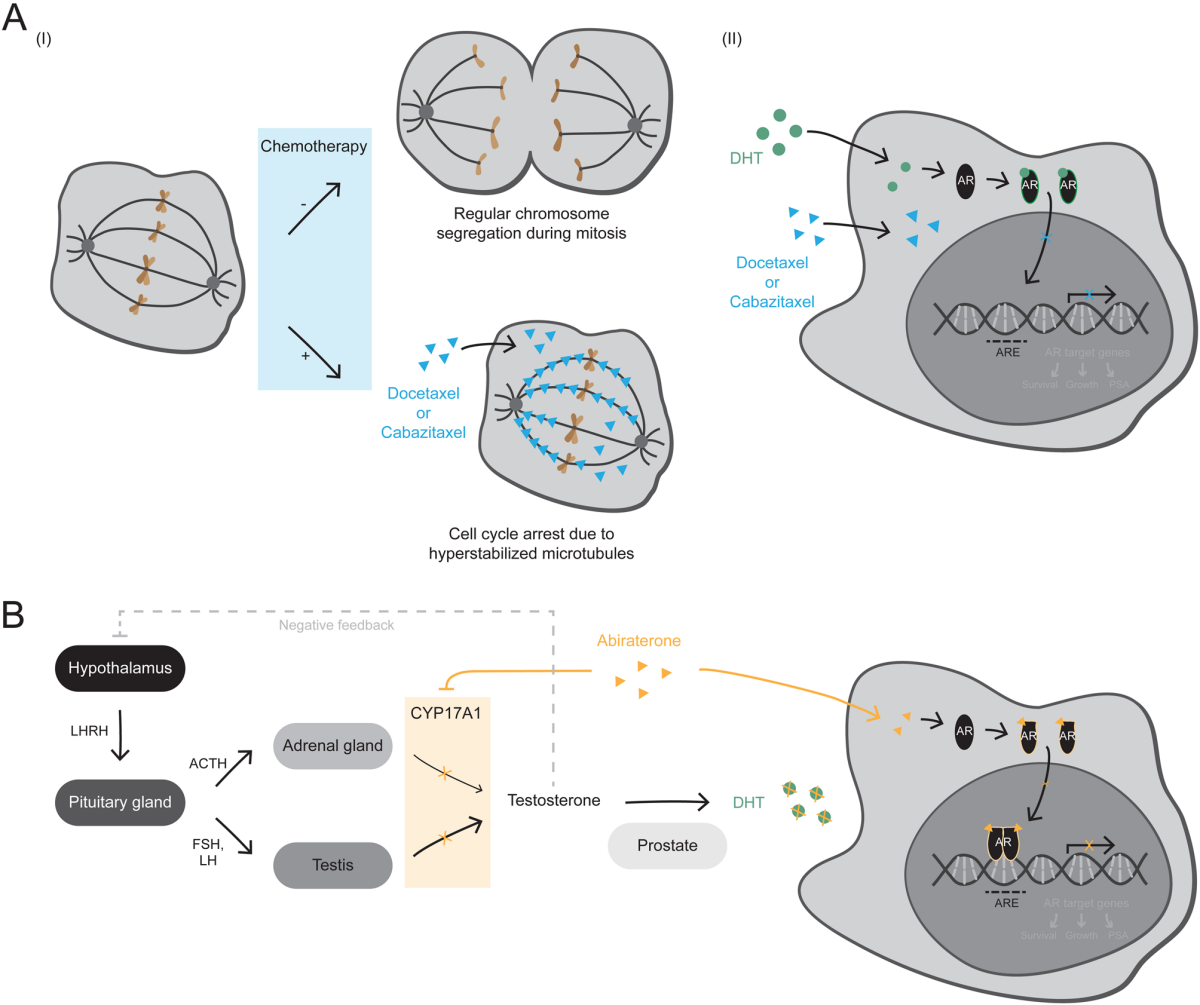
Mechanisms of action of taxane chemotherapeutics and the antiandrogen abiraterone acetate. (A) Taxane chemotherapeutics, such as docetaxel and cabazitaxel, act by hyperstabilizing microtubules, which – due to the microtubules' role in chromosome segregation during mitosis – causes a cell cycle arrest in metaphase followed by apoptosis. Moreover, taxanes directly affect AR signaling by inhibiting the microtubule-dependent AR nuclear translocation in response to androgen stimulation. (AR, androgen receptor; ARE, AR-response element; DHT, dihydrotestosterone). (B) Abiraterone is a cytochrome P450 17A1 (CYP17A1) inhibitor that leads to androgen deprivation by inhibiting the intracellular biosynthesis of androgens in the testis and adrenal glands. Androgens are produced via the hypothalamic-pituitary-testis and to a small degree also via the hypothalamic-pituitary-adrenal axis. Within these axes, CYP17A1 is responsible for converting cholesterol to androgens, such as testosterone, which gets reduced to the potent AR agonist DHT in the prostate. In addition to androgen deprivation, abiraterone is capable of directly interacting with the AR and thereby blocks the signaling of this hormone-responsive transcription factor. (ACTH, adrenocorticotropic hormone; FSH, follicle-stimulating hormone; LH, luteinizing hormone; LHRH, luteinizing hormone-releasing hormone; NRC, nuclear receptor coregulator).

Cabazitaxel is – like docetaxel – a taxane, which stabilizes microtubules and consequently impairs mitotic cell division (Fig. 4A) (Fitzpatrick and de Wit 2014, Quinn *et al.* 2017). However, the drug shows antitumor activity in docetaxel-resistant models, potentially due to the fact that cabazitaxel is a poor substrate for the drug efflux pump P-glycoprotein, which is reported to contribute to docetaxel resistance (Paller & Antonarakis 2011). In line with this, cabazitaxel has been shown to improve OS in mCRPC patients with progressive disease on or after docetaxel-based intervention (de Bono *et al.* 2010, Bahl *et al.* 2013).

#### Abiraterone acetate

Abiraterone acetate (hereafter referred to as abiraterone) is targeting the AR signaling axis by inhibiting cytochrome P450 17A1 (CYP17A1) – an enzyme involved in intracellular biosynthesis of androgens that enables prostate cancer cells to bypass androgen deprivation (Fig. 4B) (Montgomery *et al.* 2008, Attard *et al.* 2009, Helsen *et al.* 2014). In addition, it has been demonstrated that abiraterone and one of its metabolic derivatives are able to directly bind to the AR and thereby inhibit the signaling of this ligand-dependent transcription factor (Richards *et al.* 2012, Soifer *et al.* 2012, van Soest *et al.* 2013, Li *et al.* 2015). Several large clinical trials have shown its efficacy in the hormone-naïve metastatic PCa setting (Fizazi *et al.* 2017, James *et al.* 2017) as well as in the chemo-naïve (Ryan *et al.* 2015) and post-docetaxel (Fizazi *et al.* 2012) mCRPC setting.

#### Sipuleucel-T

**Figure 5:**
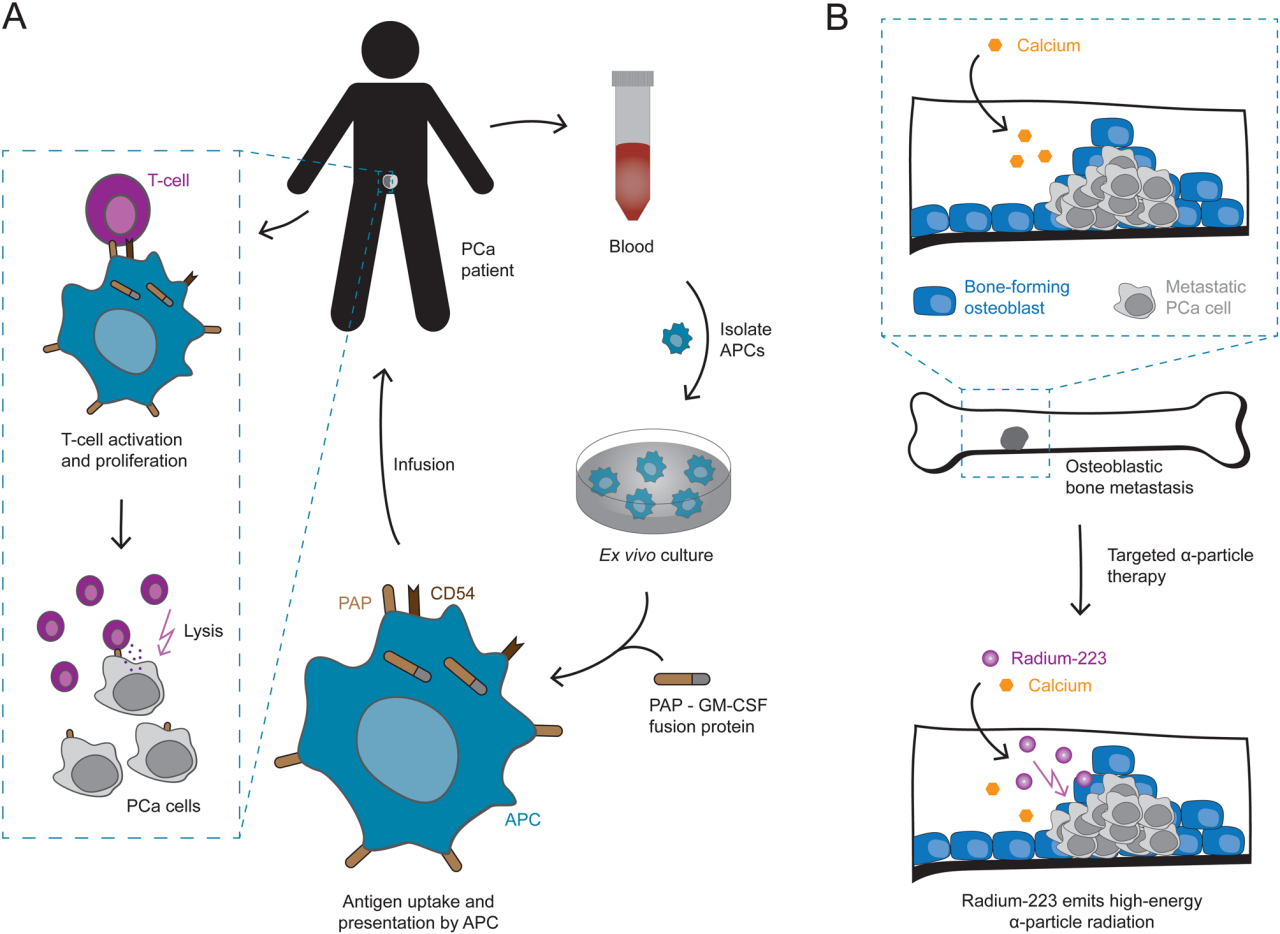
Mechanisms of action of Sipuleucel-T and Radium-223. (A) Sipuleucel-T is a cancer immunotherapy, which makes use of autologous antigen-presenting cells (APCs) to activate the patient’s immune system against PCa cells. Dendritic cells, the most efficient APCs, are isolated from blood samples and cultured *ex vivo* together with a recombinant fusion protein, composed of prostatic acid phosphatase (PAP) – an enzyme overexpressed in PCa; and granulocyte-macrophage colony-stimulating factor (GM-CSF) – a cytokine that enhances immune responses. The APCs take up these antigens, present them on their surface, and upon activation express the surface marker Cluster of Differentiation 54 (CD54) – a glycoprotein that is involved in APC – T-cell interactions. The activated APCs are then reinfused into the patient to trigger a T-cell response against PAP-expressing PCa cells. (B) Targeted alpha-particle therapy with Radium-223 is a treatment option in symptomatic mCRPC patients with skeletal metastasis. Radium-223 is a radioactive calcium analog that – like calcium itself – gets incorporated by osteoblasts into the bone matrix. This especially occurs at sites of increased bone formation, as found in bone metastasis from PCa, where Radium-223 emits high-energy alpha-particle radiation that causes severe DNA damage in nearby cells.

Sipuleucel-T is an autologous cell-based cancer immunotherapy, in which the patient’s immune system is reprogrammed to recognize and eradicate cancer cells (Higano *et al.* 2010). During the procedure, antigen-presenting cells are isolated from blood and primed *ex vivo* to recognize prostatic acid phosphatase – an enzyme overexpressed in prostate cancers – after which the activated immune cells are reinfused into the patient (Fig. 5A) (Di Lorenzo *et al.* 2011, Handy & Antonarakis 2018). In a phase III trial, this therapeutic cancer vaccine has prolonged OS of mCRPC patients with asymptomatic or minimally symptomatic disease, making it the first immunotherapeutic approach shown to improve survival in PCa (Kantoff *et al.* 2010). However, Sipuleucel-T administration has thus far only been tested with concurrent or prior to enzalutamide therapy (Nbib1981122), where both treatment schedules seem to result in similarly robust immune responses with no differences in median OS (Antonarakis *et al.* 2018, Petrylak *et al.* 2018). Until now, Sipuleucel-T is therefore considered as a therapeutic option prior to docetaxel and enzalutamide, as recommended by a European Expert Consensus Panel, unless further studies demonstrate its effectiveness in the enzalutamide-resistant mCRPC setting (Fitzpatrick *et al.* 2014).

#### Radium-223

In symptomatic mCRPC patients with skeletal metastases, Radium-223 dichloride (Radium-223) is an additional therapeutic option that improves OS (Parker *et al.* 2013). Radium-223 is a bone-seeking calcium mimetic that concentrates at areas of increased bone turnover, as found in osteoblastic bone metastases from prostate cancer, where it emits high-energy alpha-particle radiation that causes severe DNA damage in nearby cells (Fig. 5B) (Henriksen *et al.* 2002, Bruland *et al.* 2006, Parker *et al.* 2013, Deshayes *et al.* 2017).

#### Therapeutics in clinical development

The antiandrogens apalutamide (ARN-509) (Dellis & Papatsoris 2018) and darolutamide (ODM-201) (Shore 2017) are two novel therapeutics, which are currently under clinical investigation. Whereas apalutamide’s structure is highly similar to enzalutamide’s, darolutamide is structurally distinct. Nevertheless, both novel antiandrogens possess a higher affinity for the AR LBD and less passage through the blood–brain barrier compared to enzalutamide. This should reduce the risk of seizures – a common side-effect of non-steroidal antiandrogens, potentially due to an off-target binding to GABA_A_ receptors in the brain (Imamura & Sadar 2016), which in the initial phase I/II dose-escalation study occurred in about 2.1% of enzalutamide-treated patients (3 of 140), all of whom, however, received doses that were more than twice as high as the later on approved dosage of 160 mg/day (Scher *et al.* 2010, Higano *et al.* 2015). The results of a placebo-controlled phase III trial showed significantly improved metastasis-free survival and time to symptomatic progression upon apalutamide treatment in men with non-metastatic CRPC (Smith *et al.* 2018). Similarly, a study investigating the efficacy of darolutamide in this setting is presently running (Nbib2200614).

Another drug that is currently being studied with regards to overcoming enzalutamide resistance is niclosamide. It is an FDA-approved anthelminthic drug, which has been identified as a potent AR-V7 inhibitor in PCa cells, resulting in PCa cell growth inhibition *in vitro* and tumor growth inhibition *in vivo*. Further, if administered in combination with enzalutamide, niclosamide could re-sensitize enzalutamide-resistant tumors to the antiandrogen (Liu *et al.* 2014*a*). Currently, the safety and pharmacokinetics of the combination therapy are being tested in phase I trials (Nbib2532114, Nbib3123978), in which the poor oral bioavailability of niclosamide has recently been reported to limit its efficacy (Schweizer *et al.* 2018). Therefore, the current oral formulation of niclosamide might not be effective enough as a mCRPC intervention, demonstrating the importance of further clinical testing along with the development of niclosamide analogs with improved pharmacokinetic and antitumor properties (Schweizer *et al.* 2018).

Taken together, there are – at least theoretically – several alternative treatment options for mCRPC patients whose disease progressed on or after enzalutamide treatment. However, while choosing an appropriate subsequent therapeutic option, possible cross-resistance needs to be considered – especially among the next-generation antiandrogens. Moreover, a potential attenuation in a drug’s clinical efficiency may occur if used as a second- or third-line intervention, emphasizing the importance of optimal treatment scheduling.

### Optimizing the sequence of therapies

The introduction of the aforementioned novel effective therapies has added an additional dimension to the complex therapeutic landscape of mCRPC. As all of them have proven survival advantages, diverse scenarios of therapeutic interventions could be generated, but it still remains elusive how best to sequence and/or combine these treatment options.

#### Clinical and translational research exploring enzalutamide scheduling

Since it is out of the scope of this review to discuss all ongoing clinical studies with enzalutamide (co)treatment, we have compiled a non-exhaustive list of clinical trials registered on clinicaltrials.gov (Supplementary Table 1, see section on supplementary data given at the end of this article). Herein, we will focus on the limited number of studies that compare the efficacy of the various treatment options with the aim to identify an optimal sequence of treatments. One such trial is the ongoing OSTRICh study, in which patients with poor prognostic features who progressed on docetaxel therapy are randomized between cabazitaxel and either enzalutamide or abiraterone (Nbib3295565).

Sequential treatment with different AR-targeting agents has shown limited efficacy as exemplified by modest PSA responses when sequentially treated with enzalutamide and abiraterone or vice versa (Loriot *et al.* 2013, Noonan *et al.* 2013, Bianchini *et al.* 2014, Schrader *et al.* 2014, Brasso *et al.* 2015, Cheng *et al.* 2015, Petrelli *et al.* 2015, Badrising *et al.* 2016*a*). Furthermore, it is suggested that docetaxel has a reduced activity after prior therapy with enzalutamide or abiraterone (Mezynski *et al.* 2012, Aggarwal *et al.* 2013, Suzman *et al.* 2014).

A possible combinatorial treatment regimen is currently being tested in trials that evaluate the efficacy of enzalutamide in combination with taxane-based chemotherapeutics for the treatment of mCRPC. Such chemohormonal therapies have proven benefit in the metastatic non-castrate PCa setting, prior to developing hormone insensitivity. Therein, the CHAARTED (Sweeney *et al.* 2015, 2016, Kyriakopoulos *et al.* 2018) and STAMPEDE (James *et al.* 2016) trials showed that upfront addition of docetaxel chemotherapy to ADT at diagnosis of treatment-naïve metastatic PCa improves OS as compared to standard-of-care ADT. Based on these results, upfront docetaxel combined with ADT is considered to be a treatment option in men with *de novo* metastatic hormone-naïve PCa (Gillessen *et al.* 2018*a*). However, its benefit in the mCRPC setting remains elusive, as such chemohormonal combinations (such as enzalutamide + docetaxel (Nbib1565928) or enzalutamide + cabazitaxel (Nbib2522715)) have thus far only been tested in phase I/II trials with relatively small sample sizes and consequently require further study in a larger population (Morris *et al.* 2016, Sternberg 2016).

Up to now, the consensus on therapy sequencing in the mCRPC setting is mostly based on small retrospective studies that are unable to give a clear answer. Recently, a post-registration study evaluated the efficacy and safety of enzalutamide treatment in patients with mCRPC who had previously progressed on abiraterone. Therein, enzalutamide therapy was beneficial in some patients, whereas the majority presented cross-resistance between the two hormonal agents (de Bono *et al.* 2018). Similar results were shown in a retrospective study, in which the response to enzalutamide was associated with a longer interval between end of abiraterone and start of enzalutamide treatment, suggesting that over time the chance for a subsequent enzalutamide response potentially increases (Badrising *et al.* 2016*b*).

On the basis of the observed cross-resistance, it is important to evaluate which of the endocrine treatment options is more effective as first-line therapy for patients with mCRPC. This issue is currently being addressed in the ENABLE study for prostate cancer, a phase III multicenter randomized controlled trial, in which the efficacies of enzalutamide and abiraterone will be compared head to head (Izumi *et al.* 2017).

Additionally, a randomized controlled trial (Nbib2125357) is currently being performed, which assesses PSA response rates in therapy-naïve mCRPC patients being sequentially treated with abiraterone and enzalutamide or vice versa (Chi *et al.* 2017).

Combining different AR-targeting drugs simultaneously might improve efficacy as compared to consecutive treatment. This is being investigated in patients treated with enzalutamide or abiraterone alone vs a combination therapy consisting of both antiandrogens (Nbib1949337, Nbib1995513). Furthermore, although in the hormone-naïve setting, the result update of the STAMPEDE trial is awaited with high expectations, as it includes an arm with such a combination therapy (Nbib268476, Arm J).

Another approach to re-challenge enzalutamide-resistant mCRPC has been described by Schweizer *et al.* (2015) and is referred to as bipolar androgen therapy (BAT). BAT is exploiting the adaptive increase of AR levels in CRPC, allowing the tumor cells to cope with castrate levels of testosterone, by rapidly cycling between androgen stimulation and deprivation. A subsequent phase II study of BAT in mCRPC patients that progressed on enzalutamide showed successful re-sensitization to the drug, when the patients were re-challenged with the antiandrogen upon progression on testosterone therapy (Teply *et al.* 2018).

#### Current consensus guidelines for enzalutamide treatment and therapy sequencing

The St. Gallen Advanced Prostate Cancer Consensus Conference (APCCC) assists clinicians in their therapeutic decision-making regarding the management of patients with advanced prostate cancer (Gillessen *et al.* 2018*a*,*b*). The recommendations most relevant to this review have been summarized in Supplementary Table 2. Accordingly, enzalutamide is considered as a first-line treatment in patients with asymptomatic mCRPC, regardless of whether they had received ADT alone or in combination with docetaxel in the castration-sensitive setting. Similarly, enzalutamide is a first-line option for symptomatic men who received docetaxel in the castration-naïve setting; whereas either docetaxel, abiraterone or enzalutamide treatment are the therapies of choice for symptomatic patients who did not receive docetaxel in this setting. Furthermore, there was consensus that both, asymptomatic as well as symptomatic mCRPC patients, progressing on or after first-line docetaxel chemotherapy should receive either enzalutamide or abiraterone as a second-line agent.

### Novel therapeutic targets

In addition to the clinically used enzalutamide alternatives described earlier, there are currently several treatment strategies in development. The studies with the most promising (pre-) clinical data and/or ongoing clinical trials are discussed hereafter.

#### In clinical development

Recently whole-exome and transcriptome analysis of advanced PCa revealed that 89% of 150 mCRPC patients had clinically targetable aberrations (Robinson *et al.* 2015). Next to well-known frequently occurring aberrations (*AR*, *ETS*, *TP53* and *PTEN*), new genomic alterations were found to be highly enriched in mCRPC patients, including *PIK3CA/B, R-spondin, BRAF/RAF1, APC, β-catenin* and *ZBTB16/PLZF*. Furthermore, genes involved in DNA damage repair (*BRCA2*, *BRCA1* and *ATM*) were altered more frequently than expected (Robinson *et al.* 2015). More recently, Pritchard *et al.* (2016) have found that 11.8% of patients with metastatic PCa have inherited germline mutations in DNA damage repair genes, which seem to be effectively treatable with the PARP-inhibitor olaparib (Mateo *et al.* 2015). In consequence of the identification of these genomic alterations in mCRPC, there is a great interest in the design of clinical trials targeting these pathways in combination with enzalutamide treatment. Trials that are currently running include PI3K/AKT/mTOR pathway inhibition using LY3023414 (Nbib2407054), TGF-β receptor I pathway inhibition using galunisertib (Nbib2452008) and IGF1 pathway inhibition using xentuzumab (Nbib2204072).

mCRPC is also characterized by changes in the epigenetic and chromatin status like altered histone acetylation or DNA methylation, based on which chromatin readers/modifiers are regarded as potential therapeutic targets (Li *et al.* 2005, Metzger *et al.* 2005, Spans *et al.* 2016, Bennett & Licht 2018). Therefore, the BET family of proteins which recognize and bind acetylated histones and are implicated in transcriptional regulation processes are potential therapeutic targets (Padmanabhan *et al.* 2016). In particular, BRD4, a conserved member of the BET family of chromatin readers, has a crucial role in global RNA polymerase II (RNA-Pol II)-mediated transcription (Jang *et al.* 2005, Asangani *et al.* 2014). Inhibition of BRD4 recruitment to active chromatin results in displacement of RNA-Pol II from its target genes and eventually leads to growth inhibitory effects in CRPC xenograft models (Filippakopoulos *et al.* 2010, Asangani *et al.* 2014, 2016, Welti *et al.* 2018). Besides, BRD4 can physically interact with the N-terminal domain of the AR to mediate its transcriptional signaling (Yang *et al.* 2005, Asangani *et al.* 2014, Urbanucci *et al.* 2017). Hence, clinical trials have been initiated that investigate safety, pharmacodynamics, pharmacokinetics and clinical responses to a BET inhibitor (GSK525762) as monotherapy (Nbib1587703) or in combination with antiandrogens (Nbib3150056) in men with chemo-naïve or chemo-treated CRPC.

Different types of immunotherapy, e.g. anti-PD-L1 antibodies, are being examined in nearly all types of cancer, showing most efficacy in tumors with a high mutational load and an immunologically ‘hot’ tumor microenvironment (Hodi *et al.* 2010, Powles *et al.* 2014, Rizvi *et al.* 2015, Spranger 2016). However, PCa is generally characterized by a relatively ‘cold’ microenvironment with little cytotoxic T-cell infiltration (Wargo *et al.* 2016). Moreover, the mutational frequency is comparatively low, possibly restricting successful immunotherapy-mediated interventions to prostate tumors with deficient DNA damage repair (Sartor & de Bono 2018). Recently, Zehir *et al.* (2017) reported a patient case with castration- and enzalutamide-resistant PCa, who responded exceptionally well to anti-PD-L1 immunotherapy. Prospective clinical sequencing of the patient’s tumor and blood samples revealed a DNA mismatch-repair (MMR) deficiency signature in the cancerous tissue without a clear underlying somatic or germline MMR pathway lesion (Zehir *et al.* 2017). Hence, clinical trials are currently investigating the safety and efficacy of PD-L1 checkpoint inhibition as a monotherapy (using Avelumab) in patients with metastatic neuroendocrine-like PCa (Nbib3179410), and as a combinatorial treatment (using Atezolizumab and enzalutamide) in patients with mCRPC (Nbib3016312).

#### In the preclinical phase

Besides targeting the AR itself, translational research has focused over the last couple of years on finding treatment options interfering with molecules that are associated with the AR signaling pathway and thus required for proper AR action. By now, hundreds of these AR regulators and interactors have been identified, all of which could be of interest for future drug development (Heemers & Tindall 2007, Paltoglou *et al.* 2017, Stelloo *et al.* 2018). In the following, we briefly discuss therapeutic intervention strategies with promising preclinical results that target a subset of AR coregulators and thus serve as a proof of principle.

Recently, several inhibitors of the histone acetyltransferases E1A-binding protein (p300) and cAMP response element-binding protein (CREB)-binding protein (CBP) have been developed, such as GNE-049 (Jin *et al.* 2017), A-485 (Lasko *et al.* 2017) and CCS1477 (Pegg *et al.* 2017). CBP and p300 are two closely related and known transcriptional AR coactivators that have been suggested to play an important role in PCa progression (Debes *et al.* 2003, Heemers *et al.* 2007). In preclinical studies p300/CBP inhibitors block the AR transcriptional program and PCa cell proliferation in cell lines as well as castration-resistant xenograft models (Jin *et al.* 2017, Lasko *et al.* 2017, Pegg *et al.* 2017), supporting their potential clinical impact, which needs to be further validated in clinical trials. Very recently, a phase I/II trial assessing the safety and biological activity of the p300/CBP inhibitor CCS1477 as monotherapy or in combination with abiraterone or enzalutamide in mCRPC patients has been initiated (Nbib3568656).

Other AR coactivators that are currently being studied regarding their potential as therapeutic targets are the p160 steroid receptor coactivators SRC-1, SRC-2 and SRC-3 (Lonard & O’Malley 2016). SRC-1 and SRC-3 have been reported to be overexpressed in PCa cell lines and clinical specimens, where their expression levels have been associated with tumor grade and disease-specific survival (Gnanapragasam *et al.* 2001, Lonard & O’Malley 2016). Moreover, SRC-3 knockdown experiments in mice have shown decreased tumor growth, indicating its importance in prostate cancer proliferation and progression (Zhou *et al.* 2005, 2010). SRC-2 has been suggested as an PCa oncogene on the basis of integrated genomic profiling of 218 prostate tumors, illustrating *SRC-2* gene amplifications, mutations or overexpression to occur in 8% of primary and 37% of metastatic PCa lesions (Taylor *et al.* 2010). Rather recently, a novel potent small-molecule inhibitor for SRCs (SI-2) has been developed, which is setting the stage for further (pre-)clinical validation (Song *et al.* 2016). Paradoxically, not only SRC inhibition, but also hyper-stimulation can be exploited to selectively induce cancer cell death and* in vivo* tumor growth inhibition. A high-throughput screen identified a small molecule (MCB-613), which over-activates SRC transcriptional programs, leading to excessive cellular stress in cancer cells that highly rely on proper SRC functioning (Wang *et al.* 2015).

While most of the above-mentioned novel therapeutic approaches represent systemic treatments, interventions that specifically interfere with acquired features in PCa and thus would limit off-target effects, are of prime interest. A fusion of the androgen-responsive transmembrane protease serine 2 (TMPRSS2) and the v-ets erythroblastosis virus E26 oncogene homolog (ERG) is found in approximately 50% of prostate cancer cases, making it the most common genetic aberration in PCa (Tomlins *et al.* 2005, Cancer Genome Atlas Research 2015). *TMPRSS2-ERG* gene fusions lead to overexpression of the usually lowly expressed ERG master transcription factor driven by the androgen-regulated promoter of *TMPRSS2*. This is considered an early event in PCa development and phenotypically results in increased PCa cell migration, invasion and incomplete differentiation compared to benign prostate epithelial cells due to an altered transcriptional profile (Tomlins *et al.* 2008, Mounir *et al.* 2015, Kron *et al.* 2017). Thus far, three preclinical approaches have been published using either a peptide-based vaccine to prime the patient’s immune system to recognize the TMPRSS2-ERG fusion as an antigen (Kissick *et al.* 2013); liposomal nanovectors containing TMPRSS2-ERG-specific siRNAs (Shao *et al.* 2012) or cell-permeable ERG inhibitory peptides that specifically block ERG-mediated transcription by interacting with its DNA-binding domain (Wang *et al.* 2017). However, much more preclinical validations and targeting strategies have to be explored until this therapeutic approach could potentially move to the clinic.

Although AR action remains essential in mCRPC, this is not the only targetable molecule driving this complex disease. Indeed, increasing evidence suggests that a subset of antiandrogen-resistant tumors show neuroendocrine features, which seem to be a consequence of treatment-induced adaptation of adenocarcinomas with genomic and epigenomic drivers associated with decreased AR activity and epithelial plasticity (Epstein *et al.* 2014, Beltran *et al.* 2016). Efficacy of platinum-based chemotherapy has been suggested in small-cell neuroendocrine PCa before and a trial (Nbib2208583) is currently investigating this, based on the molecular phenotype of mCRPC (Aparicio *et al.* 2013).

## Conclusion and future perspectives

The introduction of enzalutamide as a second-line hormonal therapy for patients with mCRPC has led to significant improvements in the management of the disease. Due to tumor heterogeneity, the duration of benefit to enzalutamide interventions varies between patients. While some men do respond extremely well and continue treatment for several years, others progress rapidly as a result of treatment resistance. The increasing number of ongoing clinical trials reflects the successful preclinical advances in understanding enzalutamide resistance mechanisms and in discovering novel therapeutic targets to maximize clinical outcome. However, the disease continues to be terminal and current treatment options, including enzalutamide and its alternatives, have only a modest impact on survival, highlighting that many aspects of the disease remain poorly understood. Only by understanding which mechanisms underlie treatment resistance, robust molecular or clinical biomarkers can be developed to guide therapeutic decision-making and to identify patient subpopulations that benefit thereof mostly. That way, well thought-out therapeutic strategies can be designed, comprising optimal patient-tailored therapy sequencing and combination.

## Supplementary Material

Supporting Table 1

Supporting Table 2

## Declaration of interest

Van der Poel H G, Bergman A M and Zwart W receive grant support from Astellas Pharma.

## Funding

This work is supported by grants from the Alpe d’HuZes/Dutch Cancer Society KWF (10084), Movember (NKbib1), and the Dutch Organization for scientific research NWO (91716401).
