# Supplementary material for: Enzalutamide therapy for advanced prostate cancer: efficacy, resistance and beyond
Source: Endocr Relat Cancer. 2018 Sep 14;26(1):R31–52. doi: 10.1530/ERC-18-0289 (PMC6215909; doi:10.1530/ERC-18-0289)
Supplement: Supporting Table 1 [file erc-26-R31-t001.pdf]

Table S1. Non-exhaustive list of clinical trials regarding enzalutamide (co)treatment and resistance, registered on clinicaltrials.gov.

Trial and/or Registration Number	Treatment Regimen	Status
**STRIVE, NCT01664923**	Enzalutamide vs. Bicalutamide	completed
**NCT03418324**	Enzalutamide + TRC105 vs. Abiraterone + TRC105	recruiting
**NCT01547299**	Enzalutamide vs. Enzalutamide + Leuprolide + Dutasteride	completed
**ENACT, NCT02799745**	Enzalutamide vs. Active Surveillance	recruiting
**NCT02294461**	Enzalutamide vs. Placebo	active
**NCT02640534**	Enzalutamide vs. Enzalutamide + Metformin	recruiting
**PLATO, NCT01995513**	Enzalutamide + Abiraterone + Prednisone vs. Placebo + Abiraterone + Prednisone	active
**PREVAIL, NCT01212991**	Enzalutamide vs. Placebo	active
**AFFIRM, NCT00974311**	Enzalutamide vs. Placebo	completed
**ARCHES, NCT02677896**	Enzalutamide + ADT vs. Placebo + ADT	active
**PROfound, NCT02987543**	Enzalutamide/Abiraterone (+Prednisone) vs. Olaparib	recruiting
**STRIDE, NCT01981122**	Enzalutamide + Sipuleucel-T (concurrent) vs. Enzalutamide + Sipuleucel-T (sequential)	active
**NCT02407054**	Enzalutamide + LY3023414 vs. Enzalutamide + Placebo	recruiting
**NCT02607228**	Enzalutamide + GS-5829 vs. GS-5829	active
**PRESIDE, NCT02288247**	Enzalutamide + Docetaxel + Prednisolone vs. Placebo + Docetaxel + Prednisolone	active
**NCT02918968**	Enzalutamide + ADT (1st), Flutamide (2nd) vs. Flutamide + ADT (1st), Enzalutamide (2nd)	active
**NCT02685267**	Enzalutamide + Docetaxel + Prednisone vs. Docetaxel + Prednisone	active
**PROSPER, NCT02003924**	Enzalutamide vs. Placebo	active
**EMBARK, NCT02319837**	Enzalutamide vs. Enzalutamide + Leuprolide vs. Placebo + Leuprolide	recruiting
**ENZAMET, NCT02446405**	Enzalutamide + ADT vs. conventional non-steroidal antiandrogen + ADT	active
**NCT02452008**	Enzalutamide vs. Enzalutamide + LY2157299	recruiting
**NCT02012296**	Enzalutamide vs. Enzalutamide + Mifepristone	recruiting

**NCT01875250**	Enzalutamide vs. Enzalutamide + PSA-TRICOM	active
**ENZARAD, NCT02446444**	Enzalutamide + ADT + Radiotherapy vs. conventional non-steroidal antiandrogen + ADT + Radiotherapy	recruiting
**TERRAIN, NCT01288911**	Enzalutamide vs. Bicalutamide	completed
**PRIMCAB, NCT02379390**	Enzalutamide/Abiraterone (+Prednisone) vs. Cabazitaxel + Prednisone	active
**CHEIRON, NCT02453009**	Enzalutamide + Docetaxel + Prednisone vs. Docetaxel + Prednisone	unknown
**NCT02254785**	Enzalutamide/Abiraterone vs. Cabazitaxel	active
**NCT01867333**	Enzalutamide vs. Enzalutamide + PSA-TRICOM	active
**CheckMate 9KD, NCT03338790**	Enzalutamide + Nivolumab vs. Docetaxel + Prednisone + Nivolumab vs. Rucaparib + Nivolumab	recruiting
**NCT02204072**	Enzalutamide vs. Enzalutamide + BI 836845	active
**PEACE III, NCT02194842**	Enzalutamide vs. Enzalutamide + Radium-223	recruiting
**NCT02555189**	Enzalutamide vs. Enzalutamide + Ribociclib	recruiting
**NCT02125357**	Enzalutamide (1st), Abiraterone + Prednisone (2nd) vs. Abiraterone + Prednisone (1st), Enzalutamide (2nd)	active
**NCT02346578**	Enzalutamide vs. Flutamide	recruiting
**NCT02058706**	Enzalutamide + ADT vs. Bicalutamide + ADT	active
**CARD, NCT02485691**	Enzalutamide/Abiraterone + Prednisone vs. Cabazitaxel + Prednisone	recruiting
**NCT02034552**	Enzalutamide + Radium-223 vs. Abiraterone + Prednisone + Radium-223 vs. Radium-223	active
**TRITON3, NCT02975934**	Enzalutamide/Abiraterone (+Prednisone)/Docetaxel + Prednisone vs. Rucaparib	recruiting
**NCT01949337**	Enzalutamide vs. Enzalutamide + Abiraterone + Prednisone	active
**Transformer, NCT02286921**	Enzalutamide vs. Testosterone Cypionate/Enanthate	recruiting
**NCT02278185**	Enzalutamide vs. ADT	recruiting
**NCT02268175**	Enzalutamide + ADT vs. Enzalutamide + ADT + Abiraterone + Prednisone	active
**IMbassador250, NCT03016312**	Enzalutamide vs. Enzalutamide + Atezolizumab	recruiting
**PCS IX, NCT02685397**	Enzalutamide + ADT vs. Enzalutamide + ADT + Radiotherapy	recruiting

**OSTRICh, NCT03295565**	Enzalutamide/Abiraterone + Prednisone vs. Cabazitaxel	recruiting
**KEYNOTE-365, NCT02861573**	Enzalutamide + Pembrolizumab vs. Docetaxel + Prednisone + Dexamethasone + Pembrolizumab vs. Olaparib + Pembrolizumab	recruiting
**NCT03150056**	Enzalutamide + GSK525762 vs. Abiraterone + Prednisone + GSK525762	recruiting
**STAMPEDE (Arm J), NCT00268476**	Enzalutamide + Abiraterone + Prednisolone + ADT vs. Abiraterone + ADT vs. Docetaxel + Prednisolone + ADT vs. more	active
**NCT02203695**	Enzalutamide + Radiotherapy vs. Placebo + Radiotherapy	recruiting
**RE-AKT, NCT02525068**	Enzalutamide + Placebo vs. Enzalutamide + AZD5363	recruiting
**TALAPRO-2, NCT03395197**	Enzalutamide/Abiraterone (+Prednisone) + Talazoparib vs. Enzalutamide/Abiraterone (+Prednisone) + Placebo	recruiting
**NCT03568656**	Enzalutamide + CCS1477 vs. Abiraterone + CCS1477 vs. CCS1477	recruiting

The search terms used were: “enzalutamide”, study type: “interventional study”, and conditions: “prostate cancer” without time restrictions. Single-arm trials or trials without PSA progression, radiographic progression or overall survival as outcomes; and suspended, terminated or withdrawn trials were excluded from the list.
