# Supplementary material for: Enzalutamide therapy for advanced prostate cancer: efficacy, resistance and beyond
Source: Endocr Relat Cancer. 2018 Sep 14;26(1):R31–52. doi: 10.1530/ERC-18-0289 (PMC6215909; doi:10.1530/ERC-18-0289)
Supplement: Supporting Table 2 [file erc-26-R31-t002.pdf]

**Table S2. Summary of the 2017 Advanced Prostate Cancer Consensus Conference (APCCC) recommendations relevant to this review.**

| Sequence: | What is your preferred mCRPC treatment option: | Therapy: | Panel agreement: |
|---|---|---|---|
| First-line | For asymptomatic men who did not received docetaxel in the castration-naïve setting? | - Enzalutamide or Abiraterone<br>- Docetaxel<br>- Sipuleucel-T | - 86%<br>- 6%<br>- 8% |
| | For symptomatic men who did not receive docetaxel in the castration-naïve setting? | - Enzalutamide or Abiraterone<br>- Docetaxel<br>- No preferred option | - 52%<br>- 46%<br>- 2% |
| | For asymptomatic men who did receive docetaxel in the castration-naïve setting? | - Enzalutamide or Abiraterone<br>- Cabazitaxel<br>- Docetaxel<br>- Sipuleucel-T | - 90%<br>- 2%<br>- 2%<br>- 6% |
| | For symptomatic men who did receive docetaxel in the castration-naïve setting? | - Enzalutamide or Abiraterone<br>- Cabazitaxel<br>- Docetaxel<br>- Radium-223 | - 73%<br>- 19%<br>- 6%<br>- 2% |
| | For asymptomatic men who received chemohormonal therapy and progressed within ≤6 months after completion of docetaxel in the castration-naïve setting? | - Enzalutamide or Abiraterone<br>- Cabazitaxel<br>- Docetaxel<br>- Platinum-based chemotherapy<br>- No preferred option | - 77%<br>- 17%<br>- 2%<br>- 2%<br>- 2% |
| | For symptomatic men who received chemohormonal therapy and progressed within ≤6 months after completion of docetaxel in the castration-naïve setting? | - Enzalutamide or Abiraterone<br>- Cabazitaxel<br>- Platinum-based chemotherapy<br>- Radium-223<br>- Sipuleucel-T<br>- No preferred option | - 57%<br>- 27%<br>- 4%<br>- 8%<br>- 2%<br>- 2% |
| | The first-line AR pathway inhibitor should be: | - Enzalutamide | - 24% |

| | | | |
|---|---|---|---|
| | | - Abiraterone<br>- No preferred option | - 35%<br>- 37% |
| Second-line | For men with asymptomatic mCRPC who had progressive disease as best response to first-line enzalutamide or abiraterone? | - Enzalutamide or Abiraterone‡<br>- Taxane<br>- Radium-223<br>- Sipuleucel-T<br>- No preferred option | - 14%<br>- 70%<br>- 4%<br>- 6%<br>- 6% |
| | For men with symptomatic mCRPC and acquired resistance (initial response followed by progression) after use of first-line enzalutamide or abiraterone? | - Taxane<br>- Radium-223 | - 96%<br>- 4% |
| | For men with asymptomatic mCRPC who had progressive disease as best response to first-line enzalutamide or abiraterone? | - Enzalutamide or Abiraterone‡<br>- Taxane<br>- Radium-223<br>- Sipuleucel-T<br>- No preferred option | - 27%<br>- 57%<br>- 10%<br>- 4%<br>- 2% |
| | For men with symptomatic mCRPC who had progressive disease as best response to first-line enzalutamide or abiraterone? | - Taxane<br>- Radium-223<br>- No preferred option | - 90%<br>- 8%<br>- 2% |
| | For asymptomatic men progressing on or after docetaxel for mCRPC (without prior enzalutamide or abiraterone)? | - Enzalutamide or Abiraterone‡<br>- Taxane<br>- Radium-223 | - 92%<br>- 6%<br>- 2% |
| | For symptomatic men progressing on or after docetaxel for mCRPC (without prior enzalutamide or abiraterone)? | - Enzalutamide or Abiraterone‡<br>- Taxane<br>- Radium-223 | - 76%<br>- 18%<br>- 6% |
| Third-line | For men who have received enzalutamide or abiraterone as first-line treatment, and docetaxel as second-line treatment? | - Enzalutamide or Abiraterone‡<br>- Cabazitaxel<br>- Radium-223<br>- Platinum-based chemotherapy<br>- No preferred choice | - 8%<br>- 61%<br>- 15%<br>- 6%<br>- 8% |

Data extracted from the St. Gallen Advanced Prostate Cancer Consensus Conference (APCCC) manuscript (Gillessen et al. 2018a). ‡ Depending on which antiandrogen has already been used as first-line treatment.
